# Supplementary material for: Pre-/perinatal reduced optimality and neurodevelopment at 1 month and 3 years of age: Results from the Japan Environment and Children’s Study (JECS)
Source: PLoS One. 2023 Jan 11;18(1):e0280249. doi: 10.1371/journal.pone.0280249 (PMC9833583; doi:10.1371/journal.pone.0280249)
Supplement: S1 Table — aRR, adjusted relative risk (adjusted for family income and maternal education); ARTs, assisted reproductive technologies; CI, confidence interval; GDM, gestational diabetes; IUGR, intrauterine growth restriction; NDD, neurodevelopmental disorder; PIH, pregnancy-induced hypertension; SGA, small for gestational age. (DOCX) [file pone.0280249.s001.docx]

**Table S1. Adjusted relative risks of developmental concerns at 1 month for each pre/perinatal factor.**

|  | **Developmental concerns**  **at 1 month of age**  **(*n =* 14,869)** | |
| --- | --- | --- |
|  | **aRR (95% CI)** | ***p*-value** |
| **1. Young/advanced Maternal age** | 1.08 (1.05–1.12) | <0.001 |
| **2. Parity** | 2.08 (2.02–2.15) | <0.001 |
| **3. History of spontaneous abortion** | 0.94 (0.80–1.11) | 0.473 |
| **4. ARTs** | 1.25 (1.19–1.31) | <0.001 |
| **5. Threatened abortion/premature labor** | 1.04 (1.00–1.07) | 0.032 |
| **6. Antibiotic during pregnancy** | 0.98 (0.94–1.01) | 0.176 |
| **7. PIH and hypertension** | 0.97 (0.90–1.04) | 0.420 |
| **8. Psychiatric problems** | 1.07 (0.92–1.24) | 0.372 |
| **9-1. Diabetes/GDM** | 0.97 (0.90–1.05) | 0.489 |
| **9-2. Epilepsy** | 1.36 (1.10–1.68) | 0.004 |
| **9-3. Thyroidism (hyper-/hypo-)** | 1.00 (0.89–1.12) | 0.979 |
| **10. Neuropsychotropic medication use** | 1.27 (1.11–1.44) | <0.001 |
| **11. Gestational age** | 0.96 (0.92–1.00) | 0.049 |
| **12. IUGR** | 1.08 (0.98–1.18) | 0.125 |
| **13. SGA** | 1.08 (1.03–1.13) | 0.002 |
| **14. Twins or multiple birth** | 1.11 (1.01–1.22) | 0.039 |
| **15. Abnormal fetal presentations** | 0.99 (0.92–1.06) | 0.703 |
| **16. Vacuum/forceps extraction** | 1.11 (1.05–1.17) | <0.001 |
| **17. Induced delivery** | 1.02 (0.98–1.06) | 0.316 |
| **18. Cesarean section delivery** | 1.09 (1.04–1.14) | <0.001 |
| **19. Epidural analgesia** | 1.21 (1.11–1.32) | <0.001 |
| **20. Labor >24 h** | 1.06 (0.99–1.13) | 0.114 |
| **21. Apgar score (5 min)** | 1.00 (0.94–1.06) | 0.938 |
| **22. Umbilical cord/placenta problems** | 0.99 (0.96–1.02) | 0.573 |
| **23. Meconium staining** | 1.11 (1.04–1.19) | 0.003 |
| **24. Neonatal transportation** | 1.07 (1.01–1.14) | 0.028 |
| **25. Hyperbilirubinemia** | 1.05 (1.00–1.09) | 0.037 |

aRR, adjusted relative risk (adjusted for family income and maternal education); ARTs, assisted reproductive technologies; CI, confidence interval; GDM, gestational diabetes; IUGR, intrauterine growth restriction; NDD, neurodevelopmental disorder; PIH, pregnancy-induced hypertension; SGA, small for gestational age
