# Supplementary material for: Pre-/perinatal reduced optimality and neurodevelopment at 1 month and 3 years of age: Results from the Japan Environment and Children’s Study (JECS)
Source: PLoS One. 2023 Jan 11;18(1):e0280249. doi: 10.1371/journal.pone.0280249 (PMC9833583; doi:10.1371/journal.pone.0280249)
Supplement: Attachment — Submitted filename: Response to reviewers.docx [file pone.0280249.s002.docx]

4 December 2022

Dear Mr. Umberto Simeoni,

Academic Editor, PLOS ONE

Thank you for inviting us to submit our revised manuscript entitled “Pre-/perinatal reduced optimality and neurodevelopment at 1 month and 3 years of age: Results from the Japan Environment and Children’s Study (JECS)”. We also appreciate the time and effort you and the two reviewers have dedicated to providing insightful feedback on ways to strengthen our paper. It is our great pleasure to resubmit our paper for further consideration. We have incorporated changes that reflect your suggestions as follows, and hope that our edits and responses below satisfactory address all the issues and concerns you and the reviewers have noted.

Please see our responses to your comments/suggestions (written in blue).

*******************************

1. Please ensure that your manuscript meets PLOS ONE's style requirements, including those for file naming. The PLOS ONE style templates can be found at

<https://journals.plos.org/plosone/s/file?id=wjVg/PLOSOne_formatting_sample_main_body.pdf> and

<https://journals.plos.org/plosone/s/file?id=ba62/PLOSOne_formatting_sample_title_authors_affiliations.pdf>

Thank you for the information. We have incorporated the guidelines into our manuscript.

2. We note that the grant information you provided in the ‘Funding Information’ and ‘Financial Disclosure’ sections do not match.

When you resubmit, please ensure that you provide the correct grant numbers for the awards you received for your study in the ‘Funding Information’ section.

My apology for causing some confusion. As described in the end of the manuscript, the Japan Environment and Children’s Study (JECS) is funded by the Ministry of the Environment (MOE) of Japan, and the present study utilized part of the JECS data. The data collection of the JECS was funded by the MOE, and was conducted in the collaboration with the National Center, the Medical Support Center, and 15 JECS Regional Centers (Kawamoto et al., 2014), For this particular paper, the authors are fully responsible for study design, analysis, decision to publish, and preparation of the manuscript. Therefore, for this paper “Pre-/perinatal reduced optimality and neurodevelopment at 1 month and 3 years of age – Results from the Japan Environment and Children’s Study (JECS)”, "the funders had no role in study design, data analysis, decision to publish, or preparation of the manuscript.", which is what you stated in your comment 3, except regarding data collection.

Further, none of the authors received any grant or award in order to conduct this study.

3. Thank you for stating the following financial disclosure:

“The Japan Environment and Children’s Study (JECS) is funded by the Ministry of the Environment, Japan.”

Please state what role the funders took in the study.  If the funders had no role, please state: "The funders had no role in study design, data collection and analysis, decision to publish, or preparation of the manuscript."

If this statement is not correct you must amend it as needed.

Please see our comments in 2, just above this comment.

Please include this amended Role of Funder statement in your cover letter; we will change the online submission form on your behalf.

Attached please find the revised cover letter. If the content is appropriate and the corresponding author’s signature, I will have him sign and will upload a PDF file. Please let me know.

4. Thank you for stating the following in your Competing Interests section:

“No”

Please complete your Competing Interests on the online submission form to state any Competing Interests. If you have no competing interests, please state "The authors have declared that no competing interests exist.", as detailed online in our guide for authors at <http://journals.plos.org/plosone/s/submit-now>

 This information should be included in your cover letter; we will change the online submission form on your behalf.

We incorporated this point in our revised cover letter, and the sentence "The authors have declared that no competing interests exist." is now added.

5. In your Data Availability statement, you have not specified where the minimal data set underlying the results described in your manuscript can be found. PLOS defines a study's minimal data set as the underlying data used to reach the conclusions drawn in the manuscript and any additional data required to replicate the reported study findings in their entirety. All PLOS journals require that the minimal data set be made fully available. For more information about our data policy, please see <http://journals.plos.org/plosone/s/data-availability>.

Upon re-submitting your revised manuscript, please upload your study’s minimal underlying data set as either Supporting Information files or to a stable, public repository and include the relevant URLs, DOIs, or accession numbers within your revised cover letter. For a list of acceptable repositories, please see <http://journals.plos.org/plosone/s/data-availability#loc-recommended-repositories>. Any potentially identifying patient information must be fully anonymized.

Important: If there are ethical or legal restrictions to sharing your data publicly, please explain these restrictions in detail. Please see our guidelines for more information on what we consider unacceptable restrictions to publicly sharing data: <http://journals.plos.org/plosone/s/data-availability#loc-unacceptable-data-access-restrictions>. Note that it is not acceptable for the authors to be the sole named individuals responsible for ensuring data access.

We will update your Data Availability statement to reflect the information you provide in your cover letter.

Regarding the data availability, even though we chose “Yes - all data are fully available without restriction”, and it is indeed open to all the researchers registered with the JECS Core Center and the Ministry of the Environment nationwide (and we are able to provide all the information if any question regarding the analyses of this paper rises), public access is not unlimited for now unfortunately, and please copy the following paragraph for the “Data availability” section.

*Data availability:*

Data are unsuitable for public deposition due to ethical restrictions and legal framework of Japan. It is prohibited by the Act on the Protection of Personal Information (Act No. 57 of 30 May 2003, amendment on 9 September 2015) to publicly deposit the data containing personal information. Ethical Guidelines for Medical and Health Research Involving Human Subjects enforced by the Japan Ministry of Education, Culture, Sports, Science and Technology and the Ministry of Health, Labour and Welfare also restricts the open sharing of the epidemiologic data. All inquiries about access to data should be sent to: jecs-en@nies.go.jp. The person responsible for handling enquiries sent to this e-mail address is Dr Shoji F. Nakayama, JECS Programme Office, National Institute for Environmental Studies.

Source: <https://www.env.go.jp/chemi/ceh/en/index.html>

6. One of the noted authors is a group or consortium Japan Environment and Children’s Study Group. In addition to naming the author group, please list the individual authors and affiliations within this group in the acknowledgments section of your manuscript. Please also indicate clearly a lead author for this group along with a contact email address.

The list of the JECS Group members, originally in Appendix in a separate file, are now moved to the acknowledgement in the manuscript. The word “principal investigator” has been added after the name of the lead author, Michihiro Kamijima, with the contact email account.

7. Please include captions for your Supporting Information files at the end of your manuscript, and update any in-text citations to match accordingly. Please see our Supporting Information guidelines for more information: <http://journals.plos.org/plosone/s/supporting-information>.

The caption and the legend of S1 Table “S1 Table. Adjusted RRs of developmental concerns at 1 month for each pre-/perinatal factor.” have been added to the manuscript, between Disclosure and References. Please let us know if they are inserted in a wrong place. In-text citation is updated followingly (LL275-276). There is only one piece of supporting information.

Reviewers' comments:

5. Review Comments to the Author

Please use the space provided to explain your answers to the questions above. You may also include additional comments for the author, including concerns about dual publication, research ethics, or publication ethics. (Please upload your review as an attachment if it exceeds 20,000 characters)

Reviewer #1: This study using Japan's National Children's Study with over 70,000 infants and known prenatal exposures aims to understand the association between pre and peri natal exposures, parental concern for infants and developmental outcomes at 3 years.
25 perinatal states were chosen and the optimal state within each was defined. Each of the 25 items were scored 0 or 1 and appear to be weighted evenly in the analysis.
The one month parent questionnaire was based on ESSENCE shown to correlate with individuals with NDD. Score was analyzed as 0-6 or <2 >= 2

After a very detailed description of the 1 month scoring, the 3 year outcome description is much less informative. NDD were based only on parental report.

The two major concerns I have regarding this manuscript are:
1. the NDD was parental report. (this can be better described as a weakness in the manuscript)
2. The group was not adjusted for prematurity which is the driving factor in populations at risk for NDD. This weakness needs to be addressed by the authors in order to reach the conclusions that are reached.

Reviewer #2: The strengths of this study include the large sample size and rigorous statistical analysis.
However, several weaknesses also exist. Parent reported outcomes are notoriously inaccurate. The time points of 1 month and 3 years are so far apart in time that many other environmental factors likely have an impact on outcomes. More of the interim data need to be included.

Thank you very much to both reviewers for your very insightful and helpful comments. We believe that the following three points need to be improved in the manuscript: 1) limitations regarding parent-reported NDD diagnosis; 2) the time point between 1 month and 3 years of age; and 3) prematurity. Let us respond to each comment as below.

1) **Parent-reported NDD diagnosis:** We fully agree with your point, and have elaborated on the third limitation with references as follows: “Third, the information regarding NDD diagnoses was based on the parental questionnaire and was not obtained from a medical database. The reported NDDs are most likely accurate and unlikely to be overreported, because the question regarding the child’s medical history specifically asked about “diagnoses by medical doctors.” However, one cannot exclude a possibility of underreporting due to the stigma and secrecy coping followed by any NDD diagnosis at this early stage of life. (LL418-422)
2) **The time point between 1 month and 3 years of age:** We also agree with this point as we could not cover all the possible (genetic and environmental) risk factors in this study not only during pre-/perinatal periods but also after birth till three years of age. Therefore, we added the fourth limitation as follows: “Fourth, even though the pre-/perinatal periods are critical windows of neurodevelopment, not all the risk factors were incorporated in our analysis, including those during the first years of child life prior to the NDD diagnosis.” (LL422-427)
3) **Prematurity:** Thank you very much for pointing this important factor out. As we believe both pre- and post-term are risk, we included 11. Gestational age in the optimality scale, and defined that 37-40 weeks as optimal (Table 1), meaning that both pre- and post-term birth were included in one group as “non-optimal group”. Table 3 shows 10,965 participants were in the non- optimal group regarding gestational age.

Following reviewer #2’s comment, instead of stating about prematurity in the limitations, we re-categorized the participants into three (pre-term, full-term, and post-term), conducted the same analysis to calculate aRR, and added the following sentences in the manuscript (LL302-314):

“Similar to the definition of non-optimal maternal age (<20 or >35 years old), non-optimal gestational age at birth included both pre-term (<37 weeks, n = 3,448, 4.9% of the whole participants) and post-term (≥41 weeks n = 6,577, 9.2%). To investigate possible different risk of pre- and post-term birth, gestational age was recategorized into three groups: <37 weeks (pre-term), ≥37to <41 weeks (term), and ≥41 weeks (post-term). The proportion of children with NDDs in each of the three groups were 2.1%, 1.0%, and 1.3% respectively. In the bivariate analysis, crude RRs for the pre- and post-term groups for 3-year NDDs were 2.20 (95%CI 1.73-2.79, *p*<0.001), and 1.30 (95%CI 1.03 1.64, *p*<0.05) respectively. However, neither aRRs of the pre- and post-term groups in the final analysis showed statistically significant association (pre-term: aRR 0.81, 95%CI 0.56-1.17, *p*=0.259; post-term: aRR 1.21, 95%CI 0.95-1.56, *p*=0.113)”.

This change on the sub-analysis was also reflected in Statistical Analysis of the method section (LL202-206).

From the mean VIF = 1.14, we concluded that no multicollinearity between the new categorized gestational age and each of the other pre-/perinatal optimal factors was confirmed in the final analysis. No interaction between the recategorized gestational age and other factors was found as well.

Recognizing the wide range among pre-term birth (22 weeks to <37 weeks) and the fact that every day in uterus counts for premature babies, we further divided the pre-term group into three sub-groups (“extremely premature”: <28 weeks, n = 61; “very premature”: ≥28 & <32, n = 252; “moderately premature”: ≥32 & <37, n = 3,175), and found that the same tendency - the more premature they were, the higher prevalence of NDDs (and crude RR) in each group. Post-term was also shown as a risk. The crude RRs of the five groups are as follows: extremely premature 3.40 (95%CI 0.87-13.32, *p*=0.079); very premature 3.30 (95%CI 1.66-6.54, *p*=0.001); moderately premature 2.10 (95%CI 1.62 – 2.70, *p*<0.001); and post-term 1.30 (95%CI 1.02 – 1.63, *p*=0.028). As the prevalence of the NDDs was just over 1% among the whole sample at this age, and the subcategorization made each group’s number rather small, we have decided not to include the results in this manuscript while acknowledging that the gestational age at birth is one of the most important factors.

Please do not hesitate to contact me should you have any further comments/questions.

Sincerely yours,

Kahoko Yasumitsu-Lovell
